# TRPA1 and thermosensitivity

**DOI:** 10.1016/j.jphyss.2025.100010

**Published:** 2025-02-06

**Authors:** Makoto Tominaga, Moe Iwata

**Affiliations:** Thermal Biology Research Group, Nagoya Advanced Research and Development Center, Nagoya City University, Nagoya 467-8601, Japan

**Keywords:** TRPA1, Temperature, Thermosensitivity, Ion channels, Cold, Heat

## Abstract

TRPA1 was first identified as a noxious cold receptor in mice in 2003. Multiple TRPA1 genes have since been isolated, indicating that TRPA1 emerged early in evolution and showing the existence of TRPA1 variants in a range of species, including insects. Although TRPA1 channels in insects to birds (endotherms) show heat-dependent activation that indicates the importance of TRPA1 for detecting ambient warm to hot temperatures, in mammals TRPA1 temperature sensitivity remains controversial. Analyses of insect TRPA1 highlighted several important structural motifs, but the structural basis of heat-evoked activation is still unclear. Furthermore, atomic-level structures of TRPA1 solved using single particle analysis with cryo-electron microscopy did not reveal a basis for TRPA1 thermosensitivity. Recent studies did demonstrate that human TRPA1 has bimodal thermosensitivity and mouse TRPA1 is involved in noxious heat sensitivity, but additional systematic analyses are needed to determine the general mechanism of mammalian TRPA1 thermosensitivity.

## 1 Introduction

TRPA1 channels are involved in nociception as shown by multiple studies concerning a range of species from insects to humans (see reviews) [1], [2]. In insects and chickens TRPA1 functions as a heat sensor, but whether mammalian TRPA1 exhibits similar temperature sensitivity remains controversial. This review summarizes recent progress in temperature-evoked activation of TRPA1.

## 2 Identification of TRPA1

TRPA1 was first isolated in a screen for transformation-sensitive proteins in cultured fibroblasts [3]. Initially termed p120, TRPA was shown to have a similar sequence to members of the TRP channel family. In subsequent bioinformatics screening for predicted cDNA sequences containing six transmembrane (TM) domains and N-terminal ankyrin repeat domains, which are both common characteristics of TRP channels, the Patapoutian group identified a gene they termed ANKTM1 that encoded a protein having ankyrin repeat and TM domains and was predicted to be a novel thermosensitive TRP channel that they named TRPA1 [4]. In turn, full-length TRPA1 was amplified using RT-PCR from sensory neurons from mouse trigeminal ganglion (TG) and dorsal root ganglion (DRG). Mouse TRPA1 has 1125 amino acids with 14 predicted N-terminal ankyrin repeat domains followed by a 6TM domain. TRPA1 was specifically expressed in a subset of small TG and DRG neurons, particularly in peptidergic nociceptors that also express calcitonin gene-related peptide (CGRP) and TRPV1, but not TRPM8, a TRP channel that functions as a cold and menthol receptor.

Evidence that TRPA1 could function as a cold sensor came from experiments with involving Ca^2+^-imaging, whole-cell patch-clamp recording or 2-electrode voltage-clamp recording using heterologous expression of mouse TRPA1 in Chinese hamster ovary (CHO) cells or *Xenopus* oocytes [4]. The Patapoutian group showed that cold stimulus evokes an increase in intracellular Ca^2+^ concentrations and inward currents at negative potentials with an outward rectification and desensitization induced by repeated cold stimulus. The temperature thresholds were about 17 °C, a temperature that causes painful sensation in mammals, suggesting that TRPA1 functions as a noxious cold receptor. They observed two separate populations of cold-sensitive neurons in adult mouse DRG neurons: one population that appears to express TRPM8 based on responses to cool temperatures (average threshold of activation 24 °C) and to menthol, and the other population that appears to express TRPA1 based on responses to colder temperatures (average threshold of activation 15 °C), but not menthol (although menthol-dependent activation of TRPA1 was subsequently reported [5]). The latter population also included a subpopulation of capsaicin-sensitive neurons. These findings raised the possibility that painful temperatures < 17 °C are detected by TRPA1 in nociceptors and that neurons expressing TRPM8 mediate responses to innocuous cool sensations [4].

## 3 Other functions of TRPA1

Shortly after its initial identification in 2003, two groups demonstrated that TRPA1 is also a receptor for several pungent chemicals that cause painful sensations in vivo [6], [7]. The chemicals are allyl isothiocyanate that is a component of mustard oil and wasabi, which has long been used as an algesic agent to evoke nociceptive behaviors in animals, as well as cinnamaldehyde contained in cinnamon that is shown to have pro-nociceptive activity in mice. Allyl isothiocyanate and cinnamaldehyde are now used to identify TRPA1-expressing neurons in cultures of sensory neurons. TRPA1 was also shown to function downstream of activation of Gq-protein-coupled receptors like NGF and bradykinin by mediating increases in intracellular Ca^2+^ concentrations [6]. However, the Julius group observed that most (96 %) mustard oil-sensitive neurons did not have cold (5 °C) sensitivity and that TRPA1-expressing HEK293 cells did not respond to cold stimulus [7].

## 4 Is mammalian TRPA1 a cold sensor?

**Fig. 1:**
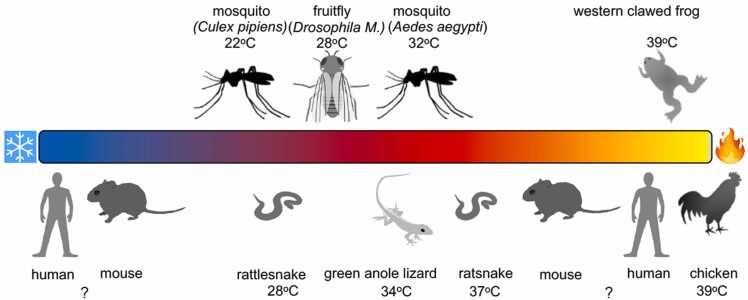
Interspecies differences in temperature sensitivities of TRPA1 orthologues. Temperatures that evoke detectable channel activation are listed.

Despite multiple studies (see reviews) [1], [2], there remains no clear consensus about whether mammalian TRPA1 acts as a cold (and/or heat) sensor (Fig. 1). Responses to cold stimuli of CHO cells, HEK293 cells or *Xenopus* oocytes expressing TRPA1 from several different species were examined using Ca^2+^-imaging, patch-clamp recording or two-electrode recordings. Responses to cold stimulus of native sensory neurons were also recorded using either rat or mouse sensory ganglia (TG, DRG or nodose ganglion to which vagal afferents project). Some reports monitored TRPA1 activity in planar lipid bilayers, which are well-suited to determine intrinsic thermosensitivity of channels like TRPA1. Alternatively, behavioral analyses were performed using wild type (WT) and TRPA1-deficient (TRPA1KO) mice to compare the effect of treatment with antagonists or antisense oligonucleotides. More systematic analyses are needed to obtain consensus for whether TRPA1 indeed functions as a cold sensor.

## 5 TRPA1KO mouse studies

Phenotypes for cold sensitivity of TRPA1KO mice were reported by two groups in 2006 that showed contrasting results. The Corey group showed that TRPA1KO mice had behavioral deficits in response to mustard oil, cold (0 °C), acetone cooling and punctate mechanical stimuli compared to those of WT mice [8]. On the other hand, the Julius group observed no difference between WT and TRPA1KO mice in acetone-evoked paw flinches, latencies for paw withdrawal or shivering in a cold-plate (〜−10 °C) assay [9]. Notably, the mice used for the behavioral analysis had a global knockout of TRPA1 expression. As such, the observed effects indicating involvement of TRPA1 in cold sensation could have been affected by loss of TRPA1 from tissues other than sensory neurons.

**Fig. 2:**
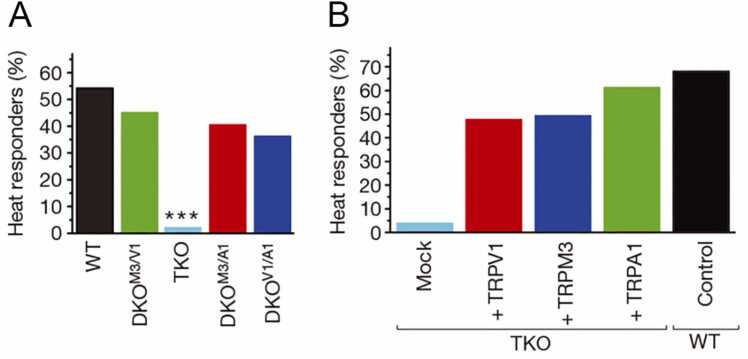
Effect of TRPA1 knockout on populations of temperature-sensitive neurons. Percentages of heat (45 °C)-responsive sensory neurons in mice with the indicated genotype (A), as well as in TKO neurons reintroduced with TRPV1, TRPM3, or TRPA1 via transfection, and WT neurons as controls (B). TKO: triple knockout. DKO: double knockout. *** p < 0.00001 vs. other genotypes (modified from Vandewauw et al. [13]) are shown.

TRPA1 as well as the thermosensitive TRP channels TRPV1 and TRPM3 have critical roles in nociception. TRPV1KO mice showed significantly longer withdrawal latency at temperatures > 50 °C compared to WT mice, but no differences at 46 °C and 48 °C in the tail immersion test, even though the temperature threshold for TRPV1 is around 43 °C in vitro [10], [11]. Similarly, TRPM3KO mice did not completely lose heat-evoked responses in a tail immersion test (45 °C −57 °C) or hot plate test (50 °C-58 °C) and did not differ from WT mice in a cold plate test [12]. The Voets group reported that mice lacking TRPV1, TRPM3 and TRPA1 (TKO (triple knockout) mice) completely lost heat-evoked pain responses, but DKO (double knockout) mice did not (Fig. 2). These results were supported by heat-evoked responses from sensory neurons isolated from TKO mice that showed a nearly complete loss of heat responses. Importantly, reintroduction of TRPV1, TRPM3 or TRPA1 via transient transfection of TKO neurons restored sensitivity to heat and to the respective channel agonists [13]. These findings indicate that initiation of an acute heat-evoked pain response in sensory nerve endings relies on three functionally redundant TRP channels to form a fault-tolerant mechanism. These studies clearly indicate the heat-sensitivity of TRPA1.

## 6 Single channel recordings

To demonstrate cold-evoked activation of TRPA1, single channel recordings in excised membrane patches or artificial lipid bilayers are essential. Using single channel recordings the Kobayashi group showed cold (5 °C)-evoked single-channel openings of mouse TRPA1 expressed in HEK293 cells in an inside/out mode, and these channels were blocked by camphor [14]. The Kim group examined single-channel currents in TRPA1-expressing HEK293 cells in a cell-attached mode. They observed cold stimulus (8 °C)-evoked single-channel openings of mouse and rat TRPA1, but not human or rhesus monkey TRPA1 in a Ca^2+^-free solution [15]. However, Ca^2+^-evoked TRPA1 activation cannot be completely ruled out even in the absence of extracellular Ca^2+^ since IP_3_ receptors in ER membranes could be in close proximity to TRPA1 in the plasma membrane.

**Fig. 3:**
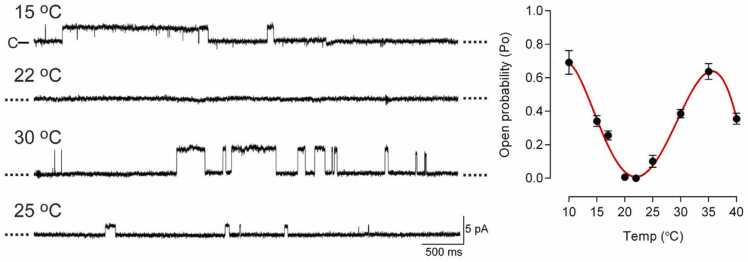
TRPA1 activity measured in a planar lipid bilayer system. Traces of single-channel recordings of human TRPA1 in planar lipid bilayers at + 60 mV (left) and the mean open probabilities (right) at different temperatures. (modified from Moparthi et al. Sci. Rep. 2016).

The Zygmunt group reported cold (10 °C)-evoked human TRPA1 activation in planar lipid bilayers that did not require the N-terminal ankyrin repeat domain [16], as well as heat (30 °C)-evoked activation of human TRPA1 using the same system [17]. Their results indicated that human TRPA1 exhibits intrinsic U-shaped thermosensitivity (Fig. 3), which could explain the apparently contrasting results reported for TRPA1 thermosensitivity. The heat-sensitivity of TRPA1 is also consistent with the findings that mice with triple knockout of TRPV1, TRPM3 and TRPA1 channels lost noxious heat sensitivity [13].

## 7 TRPA1-related symptoms in humans

Analysis of cold sensitivity in human sensory neurons is challenging, but a point mutation (N855S) in the S4 transmembrane segment of TRPA1 was identified in a genome analysis of patients with an autosomal-dominant familial episodic pain syndrome characterized by episodes of debilitating upper body pain triggered by fatigue, fasting and/or cold [18]. TRPA1 with the N855S mutation showed larger inward currents at negative potentials in response to chemical agonists or cold stimulation that was accompanied by a leftward shift in the midpoint (V_1/2_) of the voltage activation curve.

Platinum-based drugs are commonly used to treat various cancers in humans. The third-generation platinum drug oxaliplatin shows dramatic reduction in renal toxicity and ototoxicity, but exhibits a unique neurotoxic profile [19]. In addition to the chronic sensory neuropathy typical of other platinum-based drugs, oxaliplatin causes an acute syndrome, described as rapid onset of cold-exacerbated paresthesia of the hands, feet, peripheral regions and the throat that affects almost all patients. Oxaliplatin was found to evoke Ca^2+^ responses in CHO cells expressing mouse TRPA1. Moreover, oxaliplatin produced cold hyperalgesia or cold allodynia in rats and mice that were absent in TRPA1KO mice [20]. These data suggest that TRPA1 is involved in acute pain sensation and is affected by cold exposure in patients that received oxaliplatin treatment [21]. In this regard, oxaliplatin was reported to cause TRPA1 sensitization to reactive oxygen species (ROS) via inhibition of prolyl hydroxylases (PHDs). This inhibition could involve PHD-mediated hydroxylation of a proline residue within the N-terminal ankyrin repeat of human TRPA1 that endows TRPA1 with cold sensitivity through sensing of cold-evoked ROS [22]. Interestingly, TRPM3 was recently shown to be activated by heat, but not by cold in oxaliplatin-induced acute peripheral neuropathy [23].

A human randomized crossover trial with a specific TRPA1 antagonist (A-967079), involvement of TRPA1 in heat-evoked pain sensation was not confirmed [24] while the temperature threshold for cold pain appeared shifted by the inhibition of TRPA1 in the similar randomized crossover trial [25].

## 8 Species-specific temperature sensitivity of TRPA1 in ectoderms and evolution

The *Drosophila melanogaster* ortholog dTRPA1 was cloned soon after mouse TRPA1. When expressed in *Xenopus* oocytes, dTRPA1 is not activated by cold, but is activated by heat with an activation threshold between 27 °C and 29 °C [26], [27]. Heat-evoked activation of TRPA1 from mosquito (*Anopheles gambiae*) [28], [29] and the silkworm *Bombyx mori* [30] was also reported.

*In vivo* experiments demonstrated the crucial role of dTRPA1 in controlling thermotaxis and temperature preference in both larval and adult *Drosophila* (Fig. 1) [1]. The observation that the temperature threshold for activation of dTRPA1 is just above the 25 °C temperature preferred by *Drosophila* suggests that the physiological functions of dTRPA1 include both detection and subsequent avoidance of heat and noxious chemicals. Some insects use thermal cues to locate warm-blooded animals on which they feed. For these insects, the ability to differentiate between the presence of attractive warm temperatures and aversive reactive electrophiles or noxious high temperature is critical. Therefore, hematophagous animals including mosquitoes could use a strategy similar to that of TRPA1. Interestingly, it was reported that detection of thermal infrared requires the heat-activated channel TRPA1, which is expressed in neurons at the tip of the antenna of mosquito (*Aedes aegypti*) [31].

In the nematode worm *C. elegans*, TRPA1 (ceTRPA1) is expressed in various tissues. When expressed in CHO cells, ceTRPA1 exhibits mechanically activated currents. Interestingly, ceTRPA1 was reported to be activated by cold temperatures, suggesting that this channel could detect decreases in environmental temperature and contribute to an increased lifespan [32], [33]. However, a molecular phylogenetic analysis of TRPA proteins suggested that ceTRPA1 did not evolve from an ancestral TRPA1 and instead is more closely related to the basal TRPA proteins. This possibility may explain the apparent difference between ceTRPA1 and TRPA1 channels of other invertebrates.

**Fig. 4:**
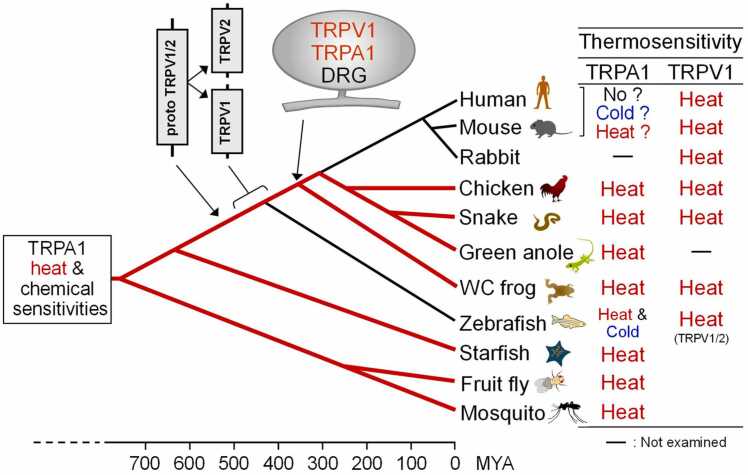
Evolutionary scenario for the nociceptive receptors TRPA1 and TRPV1 within vertebrate lineages. TRPA1 is likely to have possessed chemical and heat sensitivity early during animal evolution. The timing of gene duplication events that produced TRPV1 is not precisely known, but likely occurred, at the latest, with the most recent common ancestor of tetrapods. MYA: million years ago.

To address the question regarding cold sensitivity of TRPA1 in vertebrates, Tominaga and Saito took another approach [34], [35]. They cloned TRPA1 together with TRPV1 genes from different vertebrate species and examined the resulting temperature sensitivity as well as whether or not the functional properties of sensors involved in nociception are conserved across evolution (Fig. 4). In addition, TRPA1 and TRPV1 appear to have functional coordination since they are highly co-expressed in subsets of sensory neurons [36].

Comparison of TRPA1 channel properties revealed that TRPA1 from mammals, birds (chickens), amphibians (*Xenopus tropicalis*), and green anole lizards is activated by heat [37], [38]. This finding indicates that heat-evoked activity of TRPA1 is generally a conserved trait across a wide variety of vertebrate species. In contrast, the temperature sensitivity of TRPA1 in humans and rodents is controversial. Heterologously expressed TRPA1 from chickens (homeothermic animals) [38], reptiles (several snake species and green anole lizards), and amphibians (*Xenopus tropicalis*, *Xenopus muelleri*, *Xenopus laevis, Ra. japonica*, *Br. buergeri*, and *Br. Japonica*) is activated by heat [38], [39], [40], [41]. Meanwhile, heterologously-expressed zebrafish TRPA1 is activated by both heat and cold, but zebrafish with TRPA1KO had no behavioral abnormalities in response to either cold or heat stimulation [42]. Sensitivity of TRPA1 to electrophilic compounds such as AITC and cinnamaldehyde is also generally conserved across species. The high frequency of TRPV1 and TRPA1 co-expression in sensory neurons suggests that heat activation of TRPV1 emerged more recently together with co-expression with pre-existing TRPA1 in a subset of nociceptive neurons in ancestral vertebrates. Acquisition of TRPV1 as a novel heat sensor in ancestral vertebrates could have reduced the functional constraints of TRPA1 and contributed to changes in its thermal sensitivity in several vertebrate lineages, including mammals. Nevertheless, even these extensive analyses do not provide clear answers about events that occurred in mammals during evolution, and also do not explain the apparent cold sensitivity of TRPA1 that is observed only in mammals.

Kim et al. compared the cold sensitivity of TRPA1 in mice, rats, humans and monkeys using Ca^2+^-imaging with whole-cell or single-channel mode patch-clamp recordings. They showed that cold activates rat and mouse TRPA1, but not human or rhesus monkey TRPA1. A single residue change within the S5 transmembrane domain (G878 in rodents vs. V875 in primates) appeared to be responsible for these differences in species-specific cold activation of TRPA1 [15].

Some species developed unique physiological systems associated with thermal adaptation during the course of evolution. Several snake species, including pit vipers, boas, and pythons, acquired the ability to sense infrared radiation via a specialized organ in the head called the pit organ that is innervated by TG sensory neurons. To investigate the involvement of TRP channels in pit organs, RNA-seq analysis was carried out to compare gene expression levels between TG and DRG neurons [43], which are involved in somatosensory perception in the trunk. This transcriptome analysis revealed that TG neurons have higher TRPA1 expression levels than DRG neurons in snake species that possess a pit organ. In contrast, TRPA1 expression is similar in TG and DRG neurons from snake species that lack a pit organ. Further characterization of TRPA1 in snakes by Gracheva et al. revealed that snake species with a pit organ had a considerably lower TRPA1 temperature threshold for activation than snakes without a pit organ [43]. A detailed molecular evolutionary analysis detected signatures of positive selection in terms of an increased evolutionary rate of non-synonymous substitutions in the evolutionary lineages of snakes with pit organs [44], [45]. These results suggest that alterations in channel expression and functional properties played important roles in the acquisition of infrared detection by pit organs in snake lineages.

## 9 Factors affecting TRPA1 activity

TRPA1 has high redox sensitivity [46], [47] and can be directly activated by intracellular Ca^2+^ [48], [49]. Therefore, small changes in the redox state and/or intracellular Ca^2+^ concentration following cold exposure could cause TRPA1 activation, which might explain cold-induced TRPA1 activation. Indeed, Miyake et al. proposed that inhibition of PHD-mediated hydroxylation of human TRPA1 endows it with cold sensitivity through sensing of cold-evoked ROS [22].

## 10 Structural basis for TRPA1 heat sensitivity

Across evolution, the heat sensitivity of TRPA1 is well conserved up until mammals. Structure-function analyses of TRPA1 from various species allowed identification of several regions that are important for sensing temperature.

The thermosensitivity of *Drosophila* TRPA1 is selectively reduced due to activity in contexts in which thermosensitivity is undesirable [27] and the structural basis for this reduction is not well studied. However, the linker region is known to be important for thermosensation by *Drosophila* TRPA1 [50]. Identification of alternatively spliced TRPA1 isoforms in *Drosophila* revealed a role for the N-terminus in the regulation of heat responses [27], [50]. Strikingly, a single point mutation in the ankyrin repeat domain of mouse TRPA1 (S250N) resulted in a heat-activated channel, whereas a mutation at a homologous residue in the *Drosophila* TRPA1 (G176N) abolished heat sensitivity [51]. These data indicate the importance of N-terminal ankyrin repeats in TRPA1 temperature sensitivity. However, a TRPA1 human/*Drosophila* chimera having human N- and C-termini with the transmembrane region of *Drosophila* retains heat sensitivity, highlighting the importance of the transmembrane domain in heat sensitivity. Additional mutation analyses further supported the importance of the pore region in *Drosophila* TRPA1 for heat sensitivity [52].

Regulation of mosquito TRPA1 heat sensitivity appears to be simpler than that for *Drosophila*. The Tominaga group identified multiple alternative splice variants of the *TrpA1* gene from mosquitos in tropical regions (*Anopheles gambiae*, *Anopheles stephensi*, *Aedes aegypti*) and from temperate regions (*Culex pipiens pallens*). Comparison of TRPA1 from the four mosquito species that occupy different thermal niches revealed that TRPA1 of the temperate zone species *Culex pipiens pallens* had a lower temperature threshold for heat-evoked activation, which was supported by results of an in vivo heat-avoidance test, suggesting that TRPA1 thermosensitivity could reflect the thermal tolerance associated with the climate of the specific ecological niches [53]. Chimeric and point mutagenesis analyses revealed that two charged residues (E; glutamic acid and R; arginine) in the N-terminus ankyrin repeat in *Culex pipiens* and *Anopheles stephensi* TRPA1 are important for determining temperature thresholds. These charged amino acids likely participate in salt bridge networks involved in heat-evoked structural changes [54].

Comparison of TRPA1 from human and rattlesnake later showed clear heat sensitivity for rattlesnake TRPA1 that can be attributed to two portable heat-sensing modules within the N-terminal ankyrin repeat domain [55]. For human TRPA1, the Zygmunt group showed that purified human TRPA1 protein in planar lipid bilayers exhibited heat sensitivity that is dependent on the voltage sensing-like S1-S4 transmembrane domain and the C-terminal domain distal to the S5-S6 transmembrane segment. They also showed that the cold sensitivity of human TRPA1 is dependent on the S5-S6 pore region and the C-terminal domain [56].

## 11 Body temperature regulation by TRPA1 activity

Compounds related to thiazoline such as 2-methyl-2-thiazoline (2MT) are known to induce a potent innate fear response in mice. Furthermore, 2MT induces robust systemic hypothermia/hypometabolism and suppresses aerobic metabolism, thereby enabling long-term survival in a lethal hypoxic environment [57]. Forward genetic screening identified that freezing and avoidance behaviors induced by thiazoline-related compounds are regulated by TRPA1 in TG neurons [58]. Thiazoline-related compounds were also shown to activate TRPA1-positive sensory pathways projecting from trigeminal and vagal ganglia to the spinal trigeminal nucleus and nucleus of the solitary tract, and this activation is followed by hypothermia [59]. Although the detailed mechanisms responsible for these findings are not known, they could involve a pathway in which peripheral signal inputs sensed through TRPA1 cause hypothermia via a mechanism that is similar to that by which peripheral TRPV1 antagonism causes hyperthermia [60].

## 12 Perspective

TRPA1 plays an important physiological role in temperature sensation in a variety of species, but much remains unknown about species-specific activities of these channels. Additional work is needed to define the temperature sensitivity traits of TRPA1, particularly mammalian TRPA1. Systematic analyses of TRPA1 function in planar lipid bilayers together with characterization of in vivo phenotypes of animals having conditional TRPA1 KO are expected to reveal more details about how TRPA1 controls responses across a range temperature.

## CRediT authorship contribution statement

**Tominaga Makoto:** Writing – review & editing, Writing – original draft, Funding acquisition. **Iwata Moe:** Data curation.

## Declaration of Competing Interest

The authors declare the following financial interests/personal relationships which may be considered as potential competing interests: Makoto Tominaga reports financial support was provided by Japan Society for the Promotion of Science. If there are other authors, they declare that they have no known competing financial interests or personal relationships that could have appeared to influence the work reported in this paper.

## Data Availability

All data and materials used in the analysis are available in the manuscript or the cited works by the authors.
